# Granulomatous cheilitis associated with exacerbations of Crohn's disease: a case report

**DOI:** 10.1186/1752-1947-2-60

**Published:** 2008-02-25

**Authors:** John K Triantafillidis, Flora Zervou Valvi, Emmanouel Merikas, George Peros, Ourania Nicolatou Galitis, Aristofanis Gikas

**Affiliations:** 1 Department of Gastroenterology, "Saint Panteleimon" General Hospital, Nikea, Greece; 2 Department of Oral Medicine, "Saint Panteleimon" General Hospital, Nikea, Greece; 3 Fourth Surgical Department, University of Athens, Athens, Greece; 4 Oral Pathology and Medicine Department, School of Dentistry, University of Athens Greece; 5 Health Center of Kalivia, Attica, Greece

## Abstract

**Introduction:**

Crohn's disease is a disease involving the whole gastrointestinal tract from the mouth to the anus. Oral lesions are considered to be an important extraintestinal manifestation. Granulomatous cheilitis has been recognized as an early manifestation of Crohn's disease. It may follow, coincide with or precede the onset of Crohn's disease. The aim of this presentation is to describe a rare case of a patient with Crohn's disease in whom significant swelling of the lower lip not only preceded the diagnosis of Crohn's disease for two years, but it manifested as an early clinical index of the recurrence of the intestinal disease as well.

**Case presentation:**

A man aged 25 was admitted in our department on August 1999 with chronic diarrhea and loss of weight. His bowel symptoms started in 1998 at the age of 24. However, two years previously (June 1996) he noticed a swelling of the lower lip, which contrasted significantly with the previously normal appearance of his mouth. A lip biopsy performed at that time was compatible with granulomatous cheilitis. Crohn's disease involving the terminal ileum and large bowel was diagnosed in 1998 and confirmed on the basis of colonoscopy, enteroclysis and histology findings of the small and large bowel. Conservative treatment resulted in clinical and laboratory improvement of the bowel symptoms and lip swelling. During the following years the disease was active with exacerbations and remissions of mild to moderate severity. The swelling of the lower lip occurred in parallel with the exacerbations of the bowel disease, returning to normal during periods of remission.

**Conclusion:**

Significant swelling of the lower lip due to granulomatous cheilitis could be the first manifestation of Crohn's disease, preceding intestinal symptoms. Exacerbation of the lip lesion could be an early clinical sign of a relapse of the underlying intestinal disease.

## Introduction

Oral lesions are well-documented clinical features in patients with Crohn's disease (CD) [1-4]. The spectrum of these lesions described so far in the medical and dental literature is quite large and includes oral ulceration, labial, buccal and gingival swelling, buccal abscesses, mucosal inflammatory hyperplasia, mucosal tags and fissuring, gingivitis, granulomatous inflammation of minor salivary glands, granulomatous cheilitis [5-10], candidiasis, angular cheilitis, lichen planus, pyostomatitis vegetans, lymphadenopathy, perioral erythema, orofacial granulomatosis, midline lip fissuring, cobblestone appearance of the mucosa, and dental caries. The prevalence of oral lesions in newly diagnosed patients has been estimated to be up to 48% [1].

It is of interest that, like other extraintestinal manifestations, oral lesions may precede the onset of the underlying intestinal inflammatory disorder [3,7,8]. However, it is difficult to determine exactly which oral manifestation is certainly related to CD although it is logical to hypothesize that some of these lesions are in fact consequence of the disease or a secondary reaction to medical treatment.

Orofacial granulomatosis is a term used to describe swelling of the orofacial area, mainly the lips, secondary to an underlying granulomatous inflammatory process. Granulomatous cheilitis is the histopathological description of such inflammation occurring in the lips and surrounding tissues [5,8,9]. It has been recognized as an early manifestation of CD following, coinciding with or preceding the onset of CD [7,8]. This extraintestinal manifestation could significantly affect the quality of life of patients with CD.

Therefore, the aim of this presentation is to describe the long-term clinical course of the underlying CD in relation to the clinical behavior of the oral lesion in different time periods.

## Case presentation

**Figure 1:**
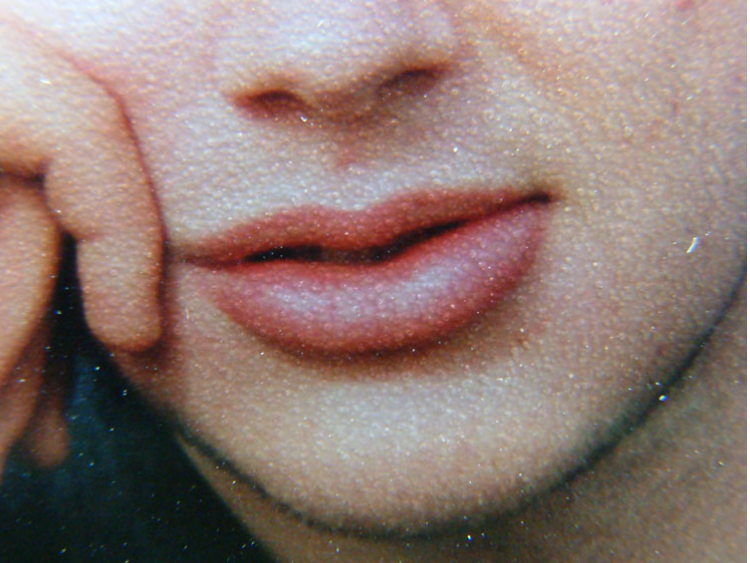
Lip swelling before the appearance of bowel symptoms (June 1996).

**Figure 2:**
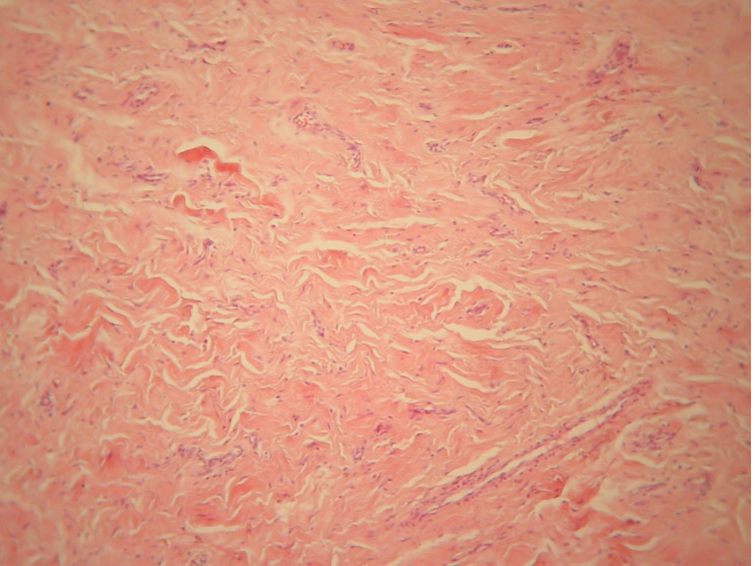
**Histological picture of the patient's lip before the diagnosis of Crohn's disease.** Multilayer squamous epithelium covering the tissue specimen, scattered clusters of lymphocytes and histiocytes resembling non-caseating granulomas, and infiltration of the small vessel walls of the underlying connective tissue by monocytes are seen.

The patient was a man aged 25. He was a smoker (20 cigarettes per day) since the age of 19. His symptoms started on June 1998 at the age of 24, with chronic diarrhea not accompanied by mucus or blood in the stools, abdominal pain, fever, or loss of weight. Two years previously on June 1996, he had noticed a slight swelling of the lower lip which contrasted with the previously normal appearance of his mouth (Figure 1). He consulted a specialist in oral medicine, who performed a lip biopsy. The histology of the specimen (13 × 7 × 7 mm) showed a multilayer squamous epithelium covering the tissue specimen, scattered clusters of lymphocytes and histiocytes resembling non-caseating granulomas, as well as infiltration of the small vessel walls of the underlying connective tissue by monocytes (Figure 2). The diagnosis was compatible with granulomatous cheilitis. Colonoscopy performed in 1998 revealed large ulcers in the terminal ileum and caecum. Enteroclysis confirmed the involvement of the terminal ileum in a total length of 50 cm. Administration of methylprednisolone, mesalazine and cholestyramine, resulted in prompt improvement of clinical symptoms and laboratory abnormalities.

**Figure 3:**
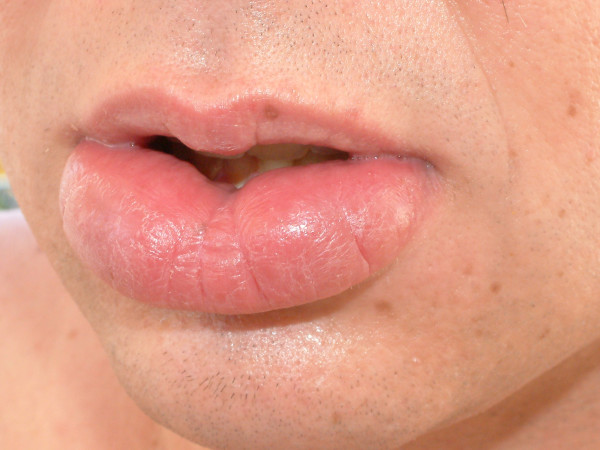
Oral lesion during exacerbation of Crohn's disease (July 2005).

During the subsequent years the disease course included at least two clinically evident recurrences of moderate severity that responded well to the administration of corticosteroids and mesalazine. Six years after diagnosis a new recurrence of CD of moderate severity (CDAI 226 points) was noticed, accompanying by lip swelling as well (Figure 3). Enteroclysis and colonoscopy plus ileoscopy showed active disease confined to the terminal ileum and the cecum.

The characteristic of the clinical course of the patient was the fact that during the exacerbations of CD, the enlargement of the lip was more evident, returning to almost normal during the periods of remission and that the administration of corticosteroids resulted in prompt improvement of the lip swelling.

## Discussion

Orofacial granulomatosis is a term generally used to describe swelling of the orofacial area, mainly the lips, secondary to an underlying granulomatous inflammatory process. It is a heterogeneous clinical condition that presents with chronic swelling of the oral or facial tissues due to granulomatous inflammation. Granulomatous cheilitis is a very rare disorder of unknown etiology, characterised by recurrent swelling of the labial tissues. The typical histological picture is the formation of scattered aggregates of non-caseating granulomas and epithelioid histiocytes. Melkersson-Rosenthal syndrome (a triad of orofacial swelling, facial paralysis and a fissured tongue) is one manifestation of orofacial granulomatosis, which more commonly presents as granulomatous cheilitis alone [5-10]. Most reported cases of orofacial granulomatosis have been in adults and some in adolescents. Orofacial granulomatosis in the paediatric population may be an initial manifestation of CD.

The interesting points regarding the clinical manifestations and the course of our patient were the fact that granulomatous cheilitis preceded the diagnosis of CD by two years, the fact that the enlargement of the lower lip occurred in parallel with the clinical exacerbations of CD and that the administration of corticosteroids resulted not only in improvement of bowel symptoms but also in diminishment of the size of the lip swelling. Another point of interest was the fact that granulomatous cheilitis was the only extraintestinal manifestation in our patient, a fact not mentioned in other descriptions.

It has been suggested that histological oral inflammation could be more common in patients with active than inactive disease [6]. According to a relevant description, patients with active CD tended to have higher scores of gingivitis than patients with inactive disease. It has also been found that smoking habit – a well established risk factor for the development of CD – and duration of disease do not have any influence on the incidence of this lesion in patients with inflammatory bowel disease.

The etiology of this lesion is unknown although various factors including levels of vitamins and trace elements, other nutritional components, and certainly the underlying inflammatory bowel disease could be involved. Mycobacterium paratuberculosis has not been found to be implicated in the pathogenesis of orofacial granulomatosis or oral CD as was suggested previously. In the absence of mechanical irritation or other mucosal disease, the oral lesions in CD could be attributed to the inflammatory bowel disease itself. It is of interest that patients with oral lesions have a significantly higher proportion of involvement of the upper gastrointestinal tract (esophagus) with CD [3,5].

Concerning treatment of granulomatous cheilitis and oral lesions of inflammatory bowel disease patients in general, it seems that topical application of corticosteroids, in conjunction with systemic treatment of the bowel disease, could be of benefit as seen in our patient [9].

This case emphasizes the fact that orofacial granulomatosis may be misdiagnosed since its clinical manifestations may be independent of or even precede the appearance of CD. Thus, patients with possible granulomatus cheilitis should be carefully asked about the presence of gastrointestinal symptoms. Those with suspicious symptoms should have a careful gastrointestinal evaluation, including enteroclysis or imaging capsule as well as complete gastrointestinal endoscopic examination. Once granulomatus cheilitis is diagnosed the patient should be followed up carefully and investigated for CD when gastrointestinal symptoms develop [6]. However, not all authors agree completely with this assumption. Van der Waal et al found a low chance of developing CD in their patients with granulomatus cheilitis and thus they suggest that patients with a negative history of gastrointestinal complaints should not be exposed to routine investigations of the gastrointestinal tract [9].

## Conclusion

From the clinical course of this patient we suggest that granulomatous cheilitis manifesting as a significant swelling of the lower lip could be the first clinical sign of CD. This lesion could well be correlated with the activity of the intestinal disease, being quite prominent in periods of exacerbation of CD and retuning to almost normal appearance during periods of remission.

## Competing interests

The author(s) declare that they have no competing interests.

## Authors' contributions

JKT drafted the paper; the patient was under his medical diagnostic and therapeutic care. FZV reviewed the histology of the lip, examined the patient, defined the exact nature of the lesion and made substantial contribution to the discussion. EM and GP critically revised the whole paper and made important suggestions. ONG reviewed the initial histology of lip and made substantial contribution to the discussion. AG contributed to the acquisition and interpretation of data and drafted the manuscript. All authors read and approved the final manuscript.

## Consent

Written informed consent was obtained from the patient for publication of this case report and accompanying images. A copy of the written consent is available for review by the Editor-in-Chief of this journal.
